# Development and validation of the Brazilian voice bank for various emotions (EMOVOX-BR)

**DOI:** 10.1590/2317-1782/e20250111en

**Published:** 2026-04-17

**Authors:** Héryka Maria Oliveira Lima, Larissa Nadjara Almeida, Alexandra Christine de Aguiar, Anna Alice Almeida

**Affiliations:** 1 Programa de Pós-graduação em Fonoaudiologia, Universidade Federal da Paraíba – UFPB - João Pessoa (PB), Brasil.; 2 Departamento de Fonoaudiologia, Universidade Federal da Paraíba – UFPB - João Pessoa (PB), Brasil.; 3 Programa de Pós-graduação em Modelos de Decisão e Saúde, Universidade Federal da Paraíba – UFPB - João Pessoa (PB), Brasil.

**Keywords:** Voice, Voice Recognition, Communication, Emotions, Behavior, Database

## Abstract

**Purpose:**

To develop and validate the Voice Bank for Various Emotions for Brazilian Portuguese (EMOVOX-BR).

**Methods:**

Observational and cross-sectional study. The corpus of this study consisted of 1,638 sound signals, in different speech tasks, produced by professional actors and actors in training, native speakers of Brazilian Portuguese (PT-BR). From these audios, those containing the phrase in PT-BR “look at the blue plane” in the variation of the six basic emotions plus neutral emission were selected. In the validation stage, the sample was composed of Brazilian speech-language pathologist judges, with experience in the area of voice, to perform the auditory-perceptual judgment of the voices to select the sound signals to compose and validate the EMOVOX-BR. They judged the identification and valence of the emotion, and which vocal parameters were most decisive in the recognition of emotions. Tests were used to verify intra- and inter-judge agreement and reliability.

**Results:**

EMOVOX-BR was made up of 39 audios, 24 male and 15 female voices. In the validation phase, all audios obtained a high accuracy rate in recognizing emotions from voice. The emotions anger, disgust and neutral were the most easily identified, with rates above 70%. The pitch and loudness parameters were the most relevant for recognizing emotions.

**Conclusion:**

EMOVOX-BR is a pioneering voice bank in PT-BR, made up of 39 audios from native speakers, with variations in the six basic emotions and neutral emission.

## INTRODUCTION

Emotions are present in the daily life of human beings and can directly affect different spheres, such as through physiological reactions, behavior and socialization. Changes in the emotional state result in a direct impact on human communication, being possible to perceive the changes in the characteristics of the voice^(1,2)^.

The voice may vary in intensity, frequency and rhythm according to emotions. Emotions interfere in the production of voice, being possible to observe its expression by personality traits, feelings, mood, among others^(3-6)^.

The relationship between voice and emotion has been widely studied over the years^(7,8)^. Some researchers evaluate universal characteristics in the recognition of emotions, and they proposed six basic emotions for being expressed in a similar way in the different cultures investigated and having specific configurations common even in the different cultures^(9,10)^.

With this, scholars from various parts of the world developed initiatives in order to form a bank of voices, to assist in the process of construction and knowledge about vocal behavior in the expression of emotions. We can highlight some international bases: Berlin Database of Emotional Speech (EMO-DB)^(11)^, Interactive Emotional Dyadic Motion Capture (IEMOCAP)^(12)^, Sustained Emotionally colored Machine-human Interaction using Nonverbal Expression (SEMAINE)^(13)^ and Remote COLaborative and Affective interactions (RECOLA)^(14)^. These are recognized in the literature and hold information about acoustic measures of speech in emotional variations.

The development and validation of a database of voices with emotional variations that holds data that involves perceptual-auditory judgment (PAJ) by speech therapy judges and speakers of Brazilian Portuguese (PT-BR) seeks to collaborate with voice perception studies, the identification of voice and speech parameters specific to each emotional state, as well as confirming that the voice can be a biological signal capable of assisting in the recognition of patterns for emotions. These findings can assist in the creation of patterns that are important for the development of human interaction systems - machine in the identification of emotions, which may cover various types of market such as call centers, applications involving voice recognition, web films, mobile communication, phonoaudiological expertise, among others^(15,16)^.

Thus, the objective of the study is to elaborate and validate the Brazilian Voice Bank in Various Emotions (EMOVOX-BR), aiming to analyze whether speech judges with experience in voice identify the emotions expressed in the audios, and which voice and speech parameters are significant in the recognition of emotions.

## METHOD

This is an observational and cross-sectional research, evaluated and approved by the Research Ethics Committee of the Center for Health Sciences of a higher education institution in Brazil, under number 3.304.419. The study was presented in two stages for better understanding: development and validation of EMOVOX-BR.

### Sample

#### Development of the EMOVOX-BR

It was based on 1,638 sound signals produced by professional actors and in training, native from several regions of Brazil and speakers of PT-BR. They simulated the six basic emotions, such as: fear, disgust, surprise, joy, sadness, anger, plus neutral emission. Three speech tasks were recorded: sustained vowel /is/, count of numbers from 1 to 10 and the phrase "look at the blue plane", sentence proposed in CAPE-V^(17)^. The latter chosen because it contains a balance of consonant and vowel sounds, which includes occlusive, fricative and diverse vowels, which favors the analysis of articulation and resonance. Its structure allows the observation of the spontaneous use of voice, prosody and fluency, while the simplicity and rhythm facilitate the modulation of intonation, intensity and speed, essential to identify vocal projection, respiratory control and emotional variation with authenticity.

Subsequently, 10 native Brazilian judges from different regions of the country, with experience in the voice area, performed the PAJ of the vocal parameters for the validation of the voice bank, selected the most suitable audios, with lower noise rate, which represented the simulated emotions. It was chosen to use the speech task composed by the phrase "Look at the blue plane", from a previous study^(18)^, which stated that the emission of balanced sentences was the best for realizing the recognition of emotions from the voice.

Thus, after this pre-analysis, the corpus of this study was made up of 200 sound signals (182 audios plus 10% repetition rate) produced by 26 professional and in-training actors, native Brazilians living in the Southeast, Northeast, South, North and Midwest of the country, with a majority of professional actors, of both sexes, with an average age of 27 (±6.75) years. All of them met the eligibility criteria: no vocal changes from the PAJ, no comorbidities that compromised cognition, hearing and communication that could limit the performance of the requested tasks; have previously answered the questionnaires selected for this survey; have access to the Internet, microphone, smartphone and/ or computer; have recorded the six variations of emotions and neutral emission, all pre-selected speech tasks.

Table 1 provides characterization data of the corpus that make up the voice bank in emotional variations.

**Table 1:** Characterization of the sample of actors and base audios for EMOVOX -BR

**Variables**	**Frequency**	**%**
**Sample**		
**Sex**		
Male	15	57.6%
Female	11	42.3%
**Education**		
Incomplete primary school	0	0%
Complete primary school	0	0%
High school	2	7.6%
Incomplete higher education	19	73%
Complete higher education	4	15.3%
Post-Graduation	1	3.8%
**Profession**		
Professional actors	16	61.53%
Performing Arts students	10	38.46%
**Participation of theatrical company**		
Yes	13	50%
No	13	50%
**Years of work**		
0-2 years	8	30.7%
3-8 years	6	23%
9-12 years	9	34.6%
More than 12 years	3	11.5%
**Brazilian region**		
Southeast	11	42.31%
Northeast	8	30.77%
South	3	11.54%
North	2	7.69%
Midwest	2	7.69%
**Audios**		
**Sex**		
Male	24	61.5%
Female	15	38.4%
**Emotion**		
Surprise	8	20.5%
Sadness	7	17.9%
Anger	7	17.9%
Neutral	7	17.9%
Fear	5	12.8%
Happiness	3	7.6%
Disgust	2	5.4%

#### Validation of the EMOVOX-BR

The sample of this stage was composed by native speech therapists from the Southeast, Northeast and South regions of Brazil, speakers of PT-BR. All judges, in addition to the training in speech therapy, had regular practice in the PAJ of vocal parameters. It was also established a minimum time of one year in the voice area, considered sufficient to develop a solid basis in analysis of vocal parameters. These criteria were adopted to ensure the reliability and validity of the judgments made by the judges in the composition of the voice bank.

All volunteers in the validation stage should follow the following eligibility criteria: be a speech therapist, have experience in the area of voice, do not have self-reported and/ or diagnosed auditory change and complete the questionnaire hosted online, with sociodemographic data and auditory-perceptual judgment of sound signals. The sample was composed of 10 speech-language and hearing judges with experience in the voice area at the validation stage (Table 2).

**Table 2:** Sociodemographic characterization and training of speech-language judges

**Variables**	**Frequency**	**Percentage**
**Education**		
Graduation	5	50%
MSc	0	0%
PhD	0	0%
Postdoctorate	5	50%
**Years since graduation**		
Less than 1 year	0	0%
1 - 5 years	4	40%
6 - 10 years	0	0%
More than 10 years	6	60%
**Specialization in the voice area**		
Yes	6	60%
No	4	40%
**Years working in the voice area**		
Less than 1 year	0	0%
1 - 5 years	4	40%
6 - 10 years	0	0%
More than 10 years	6	60%
**Brazilian region**		
Southeast	5	50%
Northeast	4	40%
South	1	10%
**Hearing disorder**		
Yes	0	0%
No	10	100%

### Materials

#### Development of the EMOVOX-BR

The tools used were: the questionnaire hosted online, with the objective of raising sociodemographic data of actors and/ or students of Performing Arts, being this composed by 12 items that addressed issues such as name, age, sex, marital status, degree of education, date and place of birth, address, e-mail, telephone, profession and family income, were also collected data about the participation in some theater company, time and period of work, in addition to investigating if the volunteer had a smartphone and/or computer and the operating system used.

The collection was carried out in a remote environment at a later time, scheduled after the response to the online form. The platform used was the Zoom Meeting video call, chosen for its practicality and easy access, and for having end-to-end data security^(18)^. The recording was done via computer and smartphone with and without the microphone of all volunteers. Also, it was used application Audacity version 3.0.2, with the use of this tool all signals were saved in "wav" format to keep the best quality, without losses, on the researcher’s computer. Three speech tasks were collected: vowel /ε/ sustained; automatic speech with counting numbers from 1 to 10; and directed speech composed by phonetic motivation phrases that make up the CAPE-V^(17-19)^. The audios selected were those related to the phrase "look at the blue plane", in the variation of emotions.

#### Validation of the EMOVOX-BR

This phase involved filling out an online form. This form contained sociodemographic data of speech and hearing judges with experience in the area of voice, consisting of 12 items that addressed the name, age, sex, marital status, date and place of birth, address, e-mail, telephone, family income, education degree, were also collected data about the time of training, if they had expertise in the area of voice and auditory alteration.

Following, the judges were instructed to listen to 200 audios in the various emotions and record the following information: the emotion identified (joy, surprise, anger, sadness, disgust, neutral and fear); the intensity or power with which the emotion was transmitted (evaluated on a scale of zero to 10); the valence (positive, negative or neutral); and the vocal parameter that they considered most relevant for emotion recognition (such as pitch, loudness, articulation, speech speed, pneumofonoarticulatory incoordination, fluency and vocal quality). Of the 200 audios, 182 were original and 18 (10%) were random repetitions, used for further analysis of intra-judge reliability.

### Data collection procedures

#### Development of the EMOVOX-BR

Initially the research was disseminated through social networks. Volunteers who showed desire to participate in the research were informed about the objectives of the research. The volunteers received instructions on the speech tasks that they had to perform and train beforehand for the simulation of the emotions in the recording session, as well as reading and agreeing with the Informed Consent Form (ICF). The ICF was also sent by e-mail with the second way signed by the main researcher.

The volunteers answered a questionnaire hosted on Google Forms. This collected sociodemographic data of actors and students of Performing Arts. After this initial collection, the volunteers received a tutorial with script and recording procedures and then performed the scheduling for the voice collection online form of the volunteers simulating the emotions. Three distinct speech tasks, mentioned above, were collected in the various emotions.

The selection of audio signals for EMOVOX-BR followed rigorous methodological and quality criteria, based on studies on online voice collection and speech tasks^(20,21)^. It was based on the use of directed speech modalities, specific to the CAPE-V protocol, and the direct capture method via line in, both recognized for ensuring a good signal-noise ratio (SNR) for remote records. To ensure the clarity and quality of the audios, all signals were submitted to an SNR analysis, with the selection restricted to audios that presented an SNR equal or greater than 30dB, according to literature standards^(22)^.

Previous studies have shown that recording with smartphones is an effective and affordable option, ensuring that voice capture occurs with satisfactory quality for further analysis^(20,21)^. Therefore, the audios collected through recording with smartphones were selected, using the Zoom meeting platform, motivated by the practicality and high quality offered by this combination in the remote environment.

The Zoom platform was selected for facilitating access to participants and providing a secure, user-friendly connection. This method ensured the inclusion of volunteers from various locations, as well as the maintenance of optimal SNR, ensuring the fidelity of the recorded signals and reducing the interference of external noises. As a result, among the 1,638 audios collected, 182 signs were chosen that met all quality and eligibility criteria. These audios represented in a reliable way the simulated emotions, being directed to the perceptual-auditory judgment performed by speech and hearing judges in the validation stage.

#### Validation of the EMOVOX-BR

In this step, we sought to collect information about the PAJ performed by the judges as well as the voice and speech parameters that were important for the recognition of emotions from the voice. The speech-language judges obtained access to the form hosted in Google Forms. This was subdivided into two sessions, initially sought to collect sociodemographic data and the second session was composed with the voices of the actors simulating the various emotions.

The judges listened carefully to the audios, evaluated and recorded their perceptions in relation to the requests described above, with the aim of identifying which audios represented each emotion most accurately. In each analysis, the judges classified the predominant emotion in each audio, considering the intensity and valence of the emotions and the most relevant vocal parameters for the recognition of the transmitted emotion. This process allowed the evaluation of the clarity and emotional consistency of each record, which ensured the representativeness of the audios in the composition of the voice bank. Each judge devoted, on average, 40 minutes to this stage of perceptual judgment, considering the total time required to evaluate all 200 audios.

### Data analysis

The data were tabulated in a digital spreadsheet for descriptive statistical analysis, by means of measures of absolute and relative frequency, as well as of central trend, such as averages and standard deviation, depending on the type of variable. Subsequently, inferential statistical tests were used. Emotions were considered as dependent variables. Valence and power of emotions and voice and speech parameters considered independent, for inferential analysis.

To identify the most representative vocal samples for each emotion, an analysis of the degree of intra-auditory reliability and inter-judge agreement was performed, using the Kappa concordance test, which is based on the number of success of the emotions proposed in each vocal sample, that is, in how many vocal samples the judges signaled the real emotion simulated by the actors.

Adequate Kappa values above 0.60 were considered, as recommended in the literature, classifying values between 0.21 and 0.39 at least; 0.40 and 0.59 weak; 0.60 and 0.79 moderate; 0.80 - 0.90 strong; above 0.90 almost perfect^(23)^.

The chi-square test was used to verify the association between the characteristics of the sample and the emotion success percentage, its valences and vocal parameters. All analyses were performed using the R software version 4.1.1.1 and the significance level of 5% was used.

## RESULTS

### Development of the EMOVOX-BR

**Figure 1:**
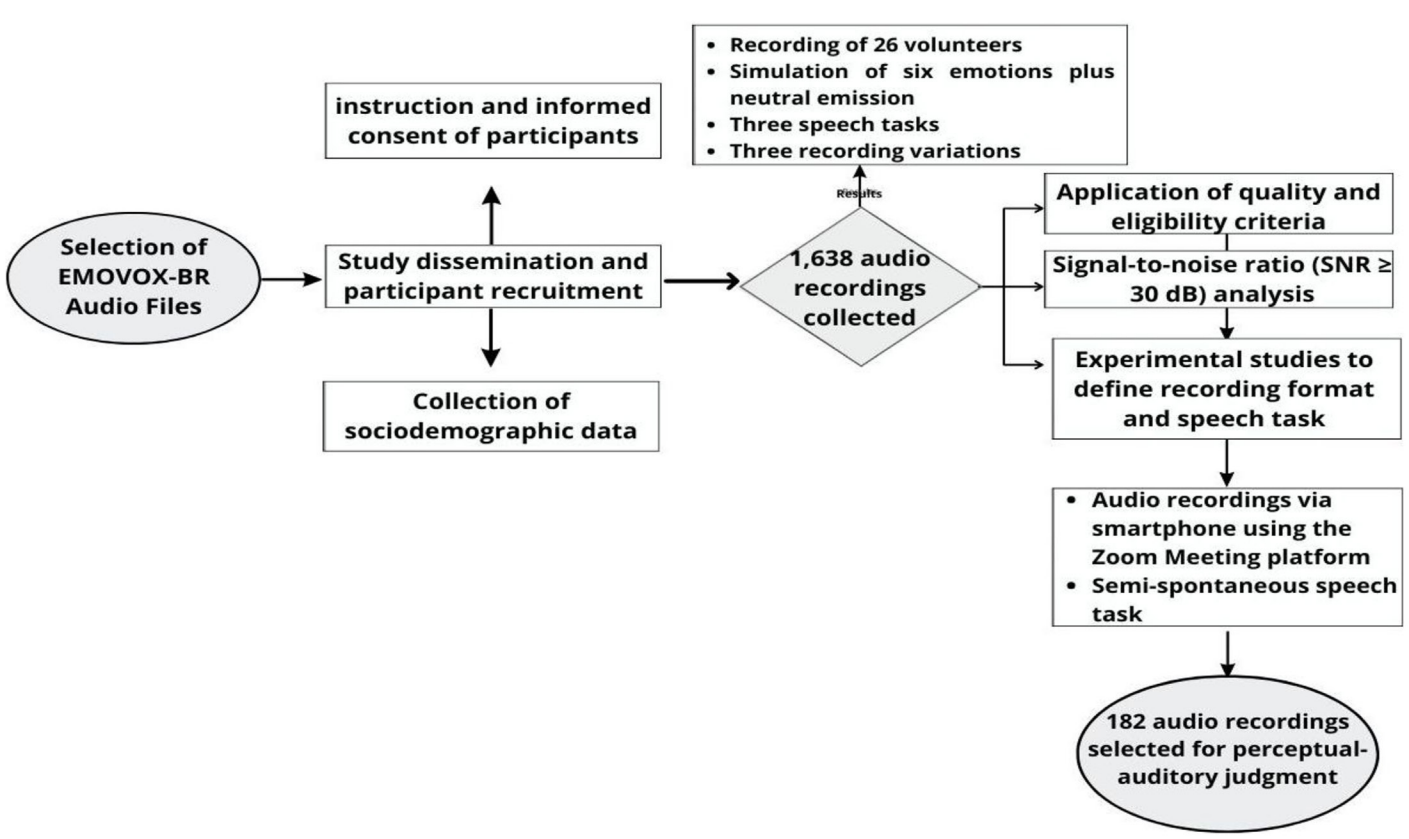
Auditory Selection Process for the auditory-perceptual judgment of EMOVOX-BR

We collected 1,638 sound signals produced by professional actors and in training whose mother language was PT-BR. Of these, 182 audios were selected to be evaluated and 39 sound signals to compose the EMOVOX-BR. Of these, 24 audios are for male voices and 15 audios for female voices. Most of the selected signals represent the surprise emotion and the smallest part the disgust emotion (Figure 1).

All 39 audios that make up the EMOVOX-BR presented reliability greater than 0.7, a value considered satisfactory according to the recommendations. The total of 24 audios (61%) obtained almost perfect agreement according to the analysis of the judges (Table 3), that is, the samples represent in fact the simulated emotion.

**Table 3:** Description of the audios and reliability value in KAPPA

**Variables**	**Sex**	**Emotion**	**KAPPA concordance value**
Audio 05	Male	Surprise	0.855
Audio 07	Male	Neutral	0.799
Audio11	Male	Anger	0.711
Audio 12	Male	Surprise	0.808
Audio 14	Male	Neutral	0.799
Audio 15	Male	Happiness	1.0
Audio 18	Male	Anger	1.0
Audio 21	Male	Neutral	1.0
Audio 26	Female	Surprise	0.714
Audio 28	Female	Neutral	0.835
Audio 30	Male	Fear	0.855
Audio 33	Male	Surprise	0.753
Audio 38	Female	Sadness	1.0
Audio 45	Male	Sadness	0.879
Audio 52	Male	Sadness	1.0
Audio 54	Male	Surprise	0.902
Audio 56	Male	Neutral	0.822
Audio 80	Male	Sadness	0.855
Audio 81	Male	Anger	0.899
Audio 82	Male	Surprise	0.783
Audio 105	Male	Neutral	0.877
Audio 121	Female	Fear	1.0
Audio 123	Female	Anger	0.799
Audio 127	Male	Happiness	0.835
Audio 128	Male	Fear	0.893
Audio 132	Male	Disgust	0.711
Audio 137	Male	Anger	0.714
Audio 138	Male	Surprise	0.783
Audio 139	Male	Disgust	1.0
Audio 142	Female	Fear	0.713
Audio 149	Female	Fear	0.855
Audio 150	Female	Sadness	1.0
Audio 151	Female	Anger	0.799
Audio 154	Female	Neutral	0.825
Audio 157	Female	Sadness	1.0
Audio 165	Female	Anger	1.0
Audio 178	Female	Sadness	0.714
Audio 180	Female	Surprise	0.808
Audio 141	Female	Happiness	0.771

### Validation of the EMOVOX-BR

The percentage of emotions in the judges' evaluation was greater than 70%, setting a high rate of recognition of emotions from the voice in the audios that make up the EMOVOX-BR bank, that is, the judges correctly identified the emotion simulated by the actors (Table 4).

**Table 4:** Success rate of the various emotions by Speech-Therapy Judges

**Emotion**	**Frequencies**	**Success Percentage**	**P-Value**
Neutral	7	84.4%	0.44*
Anger	7	82.6%	
Disgust	2	75%	
Surprise	7	73.8%	
Happiness	3	73.3%	
Sadness	7	72.5%	
Fear	5	71.3%	

Pearson’s chi-square test

* Significance p<0.05

Table 5 presents the findings regarding the perception of valence attributed by the judges for the emotions evaluated in the audios. The emotions that were defined as positive valence: joy and surprise; negative valence: fear, sadness, anger and disgust; and neutral valence: neutral. The success rate increases when the evaluation is by valence, with values above 80%. In Table 5, we observed the identification of voice and speech parameters marked as most important in the recognition of emotions presented by the judges in the PAJ.

**Table 5:** Description of the identification of the parameters of valence and voice and speech in the variation of emotions in the emission of voices according to the impressions of speech therapy judges

**Variables**	**Emotion**														**P-value**
	**Happiness**		**Fear**		**Sadness**		**Anger**		**Surprise**		**Disgust**		**Neutral**		
	**n**	**%**	**n**	**%**	**n**	**%**	**n**	**%**	**n**	**%**	**n**	**%**	**n**	**%**	
**Valency**															
Positive	27	90%	5	8.3%	1	1.3%	2	2.9%	70	85.7%	4	20%	2	2.9%	0.0001*
Negative	3	10%	51	85%	61	76.3%	63	90%	9	11.3%	16	80%	12	17.1%	0.0001*
Neutral	0	0%	4	6.7%	18	22.5%	5	7.1%	1	1.3%	0	0%	56	80%	0.0001*
**Voice and Speech parameters**															
Pitch	21	70%	30	51.7%	49	61.3%	40	57.1%	62	77.5%	13	65%	36	51.4%	0.003*
Loudness	11	36.7%	28	46.7%	28	35%	35	50%	31	38.8%	5	25%	17	24.3%	0.003*
Articulation	4	13.3%	4	6.7%	3	3.8%	17	24.3%	10	12.5%	4	20%	5	7.1%	0.0002*
F Speed	11	36.7%	18	30%	33	41.3%	33	47.1%	28	36.3%	70	35%	24	34.3%	0.004*

Pearson’s chi-square test

* Significance p<0.05

It is observed that the pitch was the most cited parameter to identify all basic emotions: joy, fear, sadness, anger, surprise and disgust. Loudness was important to recognize anger emotion. Articulation, speech speed, PFAIC, vocal quality and fluency were not important parameters to determine the classification of the presented emotions in all emotions with a result lower than 50%.

## DISCUSSION

The elaboration and validation process of the pioneer EMOVOX-BR arose from the scarcity of voices with emotional variations in the PT-BR, as well as the novelty of passing through the validation process by speech-therapy judges with experience in the voice area. EMOVOX-BR was composed of 39 audios, of which 24 are male voices and 15 female voices, covering the emotions joy, surprise, anger, sadness, disgust, neutral and fear. The selection of the six basic emotions, recognized by "Big-Six", plus the neutral emission, which refers to the audio signal that has no predominance of any of the emotions, was directed from previous studies^(18,20,21,24)^.

Currently, several voice banks incorporate emotional variations and cover populations of actors in different languages and cultures around the world, but few are available for open access to the scientific community^(11-14)^. Most of these banks were developed with samples of adult speakers, and only two include children’s voices, one of which is specific for stress variations.

Some of these banks share a similar development structure, which includes characteristics such as participant selection, accessibility and types of emotions analyzed^(12-14)^. However, these internationally recognized bases were created primarily to investigate acoustic speech variations, with little emphasis on PAJ by experts^(11)^. In addition, they are international bases and do not include samples of PT-BR speakers.

Some parameters identified in the voice and speech can be understood as a set of suprassegmental phenomena, such as the speed of speech, rhythm in the temporal aspect, melodic organization (accent, melody, intonation) and intensity (volume, strength) present in speech^(25,26)^, as well as the psychoacoustic parameters, pitch and loudness.

PAJ is a traditional resource used in the clinical practice of the voice area, but it depends on the experience of the evaluator^(27-29)^. The course of speech therapy prepares for the judgment of vocal parameters, especially those related to vocal quality. The literature indicates, however, that in addition to intensive training, other factors are essential to increase the reliability of judges in auditory perception, such as exposure to a wide variety of voices, quality and diversity of the vocal material used, the type of question asked and previous experience with different emotional nuances^(28,29)^.

The accuracy percentage of the emotions evaluated by the judges in the signals that make up EMOVOX-BR indicates that the vocal samples are representative and effective in the expression of emotions. Surprise emotion, followed by sadness and anger, presents the highest number of audios selected for EMOVOX-BR, while disgust has the lowest number of audios. The emotions with the highest percentage of success were anger and neutral, while fear had the lowest rate of success. This finding is confirmed by the literature, which highlights anger as the emotion of greater impact in the identification by the interlocutor, requires more energy for its production and can be associated with changes in larynx positioning, speech speed and vocal intensity^(10,20,27)^.

There was a high success rate in the recognition of emotions from the voice by the group of professionals with experience in the area of voice, where they obtained values greater than 70% in all emotions (joy, fear, sadness, anger, surprise, disgust and neutral). Previous study^(30)^ on low and high anxiety detection in voice analysis with lay professionals and generalists obtained a success rate around 50%, so the experience of judges may be a finding that influences the success rate.

The valence of emotions, analyzed in this study, refers to the positive, negative or neutral character of expressed emotion^(31)^. In addition to the positive and negative valences, the neutral valency was also considered, characterized by the absence of a specific emotion in vocal emission. The judges classified the emotions as positive (joy and surprise), negative (fear, anger, sadness and disgust) and neutral (neutral). It is observed that changes in emotions and valences influence vocal expressiveness, and the repetition of negative emotional patterns can result in physiological effects, such as dryness of the oral mucosa, which may interfere with speech, with repercussions on oral muscle control and phoneme articulation, in addition to possibly causing vocal quality instability^(7,32)^. Thus, the emotional state affects the communicative activity as a whole, and not only the voice^(30)^.

The identification of voice and speech parameters was essential for the characterization of emotions in the auditory, according to the PAJ performed by speech and hearing judges. It was observed that the pitch was the parameter most often associated with emotions joy, fear, sadness, anger, surprise, disgust and neutral, while loudness stood out in emotion anger. Other parameters, such as articulation, speech speed, fluency, vocal quality and pneumofonoarticulatory incoordination (PFAIC), were not considered relevant for the classification of emotions. This finding can be explained by the absence of vocal alterations in speakers, whose signals did not present dysphonia characteristics that could compromise the intelligibility and vocal expressiveness.

It is important to highlight that the pitch and loudness are psychoacoustic parameters, because they depend on the perception of the listener, with pitch associated with the frequency of sound and loudness to perceived intensity. In the context of this study, these parameters help to identify emotional nuances, which may allow exploring the emotional load of the voice beyond vocal quality, which is essential to capture the complexity of expressiveness^(33)^.

In general, the pitch and loudness parameters were reaffirmed as fundamental for the identification of emotions from the voice^(7-9,32)^. Studies indicate that, in different languages, joy of emotion is often characterized by a high pitch, strong loudness and brief pauses. Anger emotion, in turn, features high pitch, weaker loudness in men and stronger in women, plus a faster speaking speed in women. Already the disgust emotion is commonly expressed by a low pitch, weak loudness and reduced speech speed, which reflects its intensity and negative valence^(34,35)^.

Therefore, experienced judges were able to recognize the emotions from the voice in native speakers of PT-BR and perceive common characteristics in emotional variations through the PAJ. These findings contribute to the scientific community since they have audios tested and validated with high reliability, which can assist in the creation of pattern recognition systems, and in the speech therapy clinic can help in the deepening of future studies on strategies of self-regulation, behavior change, vocal control and management of emotions.

More possibilities open in a perspective focused on vocal improvement, psychodynamics and interventions aimed at voice professionals, such as composition of characters with actors. In addition, it confirms that the voice is a biological signal of interdisciplinary interest, collected in a non-invasive way, which opens several possibilities for studies of pattern recognition using cutting-edge statistical models to discriminate or predict various technological contexts, social, cultural and health.

## CONCLUSION

The Brazilian Voice Bank in Varied Emotions (EMOVOX-BR) was developed and validated to represent the emotions joy, fear, sadness, anger, surprise, disgust and neutral in speakers of PT-BR. Composed by 39 audios of high reliability in terms of quality, identification and intensity of emotions, the bank obtained a high success rate in the evaluation of judges with experience in the area of voice. The pitch parameter stood out in identifying all emotions, while loudness was particularly relevant to identify anger emotion. Thus, the EMOVOX-BR was validated, a fact that demonstrates its effectiveness in representing different emotions and their distinctive characteristics, perceptible by specialized evaluators.
