# Supplementary material for: Development and validation of the Brazilian voice bank for various emotions (EMOVOX-BR)
Source: Codas. 2026 Apr 17;38(3):e20250111. doi: 10.1590/2317-1782/e20250111en (PMC13142256; doi:10.1590/2317-1782/e20250111en)
Supplement: Translation

# Translation: Elaboração e validação do banco de vozes brasileiro nas variadas emoções (EMOVOX-BR)

**DOI:** 10.1590/2317-1782/e20250111pt

**Authors:** Héryka Maria Oliveira Lima, Larissa Nadjara Almeida, Alexandra Christine de Aguiar, Anna Alice Almeida

**Affiliations:** 1 Programa de Pós-graduação em Fonoaudiologia, Universidade Federal da Paraíba – UFPB - João Pessoa (PB), Brasil.; 2 Departamento de Fonoaudiologia, Universidade Federal da Paraíba – UFPB - João Pessoa (PB), Brasil.; 3 Programa de Pós-graduação em Modelos de Decisão e Saúde, Universidade Federal da Paraíba – UFPB - João Pessoa (PB), Brasil.

**Keywords:** Voz, Reconhecimento de Voz, Comunicação, Emoções, Comportamento, Banco de Dados

## Abstract

**Objetivo:**

Elaborar e validar o Banco de Vozes nas Variadas Emoções para o português brasileiro (EMOVOX-BR).

**Método:**

Estudo observacional e transversal. O corpus deste estudo foi constituído por 1.638 sinais sonoros, em diferentes tarefas de fala, produzidos por atores profissionais e em formação, nativos e falantes do português brasileiro (PT-BR). Desses áudios, selecionou-se os que continham a frase em PT-BR “olha lá o avião azul” na variação das seis emoções básicas mais emissão neutra. Na etapa de validação, a amostra foi composta por juízas fonoaudiólogas brasileiras, com experiência na área de voz, para realizar o julgamento perceptivo-auditivo das vozes para selecionar os sinais sonoros para compor e validar o EMOVOX-BR. Julgaram a identificação e valência da emoção, e quais parâmetros vocais foram mais decisivos no reconhecimento das emoções. Utilizou-se testes para verificar a concordância e confiabilidade intra e interjuizas.

**Resultados:**

O EMOVOX-BR foi formado por 39 áudios, 24 vozes masculinas e 15 femininas. Na fase de validação, todos os áudios obtiveram uma alta taxa de acerto no reconhecimento das emoções a partir da voz. As emoções, raiva, nojo e neutra foram as mais facilmente identificadas, com taxas superiores a 70%. Os parâmetros pitch e loudness foram os mais relevantes para o reconhecimento das emoções.

**Conclusão:**

O EMOVOX-BR é um banco de vozes pioneiro no PT-BR, composto por 39 áudios de falantes nativos, com variação nas seis emoções básicas e emissão neutra.

## INTRODUÇÃO

As emoções estão presentes no cotidiano dos seres humanos e podem repercutir diretamente em diferentes esferas, como por meio de reações fisiológicas, comportamentais e na socialização. Mudanças do estado emocional resultam em um impacto direto na comunicação humana, sendo possível perceber as mudanças nas características da voz^(1,2)^.

A voz pode apresentar variações de intensidade, frequência e ritmo, de acordo com as emoções. Percebe-se que as emoções interferem na produção da voz, sendo possível observar sua expressão por traços de personalidade, sentimentos, humor, dentre outros^(3-6)^.

A relação entre voz e emoção vem sendo amplamente estudada ao longo dos anos^(7,8)^. Há pesquisas que avaliam características universais no reconhecimento das emoções, e propuseram seis emoções básicas por serem expressas de forma semelhante nas diferentes culturas investigadas e possuírem configurações específicas comuns mesmo nas diferentes culturas^(9,10)^.

Com isso, estudiosos de várias partes do mundo desenvolveram iniciativas com intuito de formar banco de vozes, para auxiliar no processo de construção e conhecimento acerca do comportamento vocal na expressão das emoções. Pode-se destacar algumas bases internacionais: Berlin Database of Emotional Speech (EMO-DB)^(11)^, Interactive Emotional Dyadic Motion Capture (IEMOCAP)^(12)^, Sustained Emotionally colored Machine-human Interaction using Nonverbal Expression (SEMAINE)^(13)^ e Remote COLaborative and Affective interactions (RECOLA)^(14)^. Essas são reconhecidas na literatura e detém informações acerca de medidas acústicas da fala nas variações emocionais.

A elaboração e validação de um banco de vozes com variações emocionais que detenha dados que envolve o julgamento perceptivo-auditivo (JPA) por parte de juízes fonoaudiólogos e falantes do Português Brasileiro (PT-BR) busca colaborar com os estudos de percepção da voz, a identificação de parâmetros de voz e fala específicas de cada estado emocional, além de ratificar que a voz pode ser um sinal biológico capaz de auxiliar no reconhecimento de padrões para as emoções. Esses achados podem auxiliar na criação de padrões que são importantes para o desenvolvimento de sistemas de interação homem - máquina na identificação de emoções, que poderá abarcar diversos tipos de mercado como *call center*, aplicativos que envolvem reconhecimento de voz, web filmes, comunicação móvel, perícia fonoaudiológica, entre outros^(15,16)^.

Assim, o objetivo do estudo é elaborar e validar o Banco de Vozes Brasileiro nas Variadas Emoções (EMOVOX-BR), com a finalidade de analisar se juízes fonoaudiólogos com experiência em voz identificam as emoções expressas nos áudios, e quais os parâmetros de voz e fala são marcantes no reconhecimento das emoções.

## MÉTODO

Esta é uma pesquisa observacional e transversal, avaliada e aprovada pelo Comitê de Ética em Pesquisa do Centro de Ciências da Saúde de uma instituição de ensino superior do Brasil, sob número 3.304.419. O estudo foi apresentado em duas etapas para melhor compreensão: elaboração e validação do EMOVOX-BR.

### Amostra

#### Elaboração do EMOVOX-BR

Partiu-se de 1.638 sinais sonoros produzidos por atores profissionais e em formação, nativos de diversas regiões do Brasil e falantes do PT-BR. Eles simularam as seis emoções básicas, tais como: medo, nojo, surpresa, alegria, tristeza, raiva, além da emissão neutra. Foram gravadas três tarefas de fala: vogal sustentada /é/, contagem de números de 1 a 10 e a frase "olha lá o avião azul", frase proposta no CAPE-V^(17)^. Essa última escolhida por conter um equilíbrio de sons consonantais e vocálicos, que inclui oclusivos, fricativos e vogais diversas, o que favorece a análise de articulação e ressonância. Sua estrutura permite observar o uso espontâneo da voz, prosódia e fluência, enquanto a simplicidade e ritmo facilitam a modulação de entonação, intensidade e velocidade, essenciais para identificar projeção vocal, controle respiratório e variação emocional com autenticidade.

Posteriormente, 10 juízas nativas brasileiras de diferentes regiões do país, com experiência na área de voz, realizaram o JPA dos parâmetros vocais para a validação do banco de vozes, selecionaram os áudios mais adequados, com menor taxa de ruído, que representaram as emoções simuladas. Optou-se por utilizar a tarefa de fala composta pela frase “Olha lá o avião azul”, a partir de estudo prévio^(18)^, que afirmou que a emissão de frases balanceadas foi a melhor para realização do reconhecimento das emoções a partir da voz.

Assim, após essa pré-análise, o *corpus* deste estudo foi constituído por 200 sinais sonoros (182 áudios mais 10% de taxa de repetição) produzidos por 26 atores profissionais e em formação, nativos brasileiros residentes das regiões Sudeste, Nordeste, Sul, Norte e Centro-Oeste do país, sendo maioria atores profissionais, de ambos os sexos, com média de idade de 27 (±6,75) anos. Todos eles atenderam aos critérios de elegibilidade: não apresentar alterações vocais a partir do JPA, não apresentar comorbidades que comprometiam a cognição, audição e comunicação que pudesse limitar a realização das tarefas solicitadas; ter respondido previamente aos questionários selecionados para esta pesquisa; ter acesso à internet, microfone, smartphone e/ou computador; ter realizado as gravações nas seis variações das emoções e na emissão neutra, de todas as tarefas de fala pré-selecionadas.

A Tabela 1 fornece dados de caracterização do *corpus* que compõem o banco de vozes nas variações emocionais.

**Tabela 1:** Caracterização da amostra de atores e áudios base para o EMOVOX -BR

**Variáveis**	**Frequência**	**Porcentagem**
**Amostra**		
**Sexo**		
Masculino	15	57,6%
Feminino	11	42,3%
**Grau de Instrução**		
Fundamental incompleto	0	0%
Fundamental completo	0	0%
Ensino médio	2	7,6%
Ensino superior incompleto	19	73%
Ensino superior completo	4	15,3%
Pós-Graduação	1	3,8%
**Profissão**		
Atores profissionais	16	61,53%
Estudantes de Artes Cênicas	10	38,46%
**Participação de companhia teatral**		
Sim	13	50%
Não	13	50%
**Tempo de atuação**		
0-2 anos	8	30,7%
3-8 anos	6	23%
9-12 anos	9	34,6%
Maior que 12 anos	3	11,5%
**Região do Brasil**		
Sudeste	11	42.31%
Nordeste	8	30.77%
Sul	3	11.54%
Norte	2	7.69%
Centro-oeste	2	7.69%
**Áudios**		
**Sexo**		
Masculino	24	61,5%
Feminino	15	38,4%
**Emoção**		
Surpresa	8	20,5%
Tristeza	7	17,9%
Raiva	7	17,9%
Neutra	7	17,9%
Medo	5	12,8%
Alegria	3	7,6%
Nojo	2	5,4%

#### Validação do EMOVOX-BR

A amostra desta etapa foi composta por fonoaudiólogas nativas das regiões Sudeste, Nordeste e Sul do Brasil, falantes do PT-BR. Todas as juízas, além da formação em Fonoaudiologia, possuíam prática regular no JPA de parâmetros vocais. Estabeleceu-se, ainda, um tempo mínimo de um ano de atuação na área de voz, considerado suficiente para desenvolver uma base sólida em análise dos parâmetros vocais. Esses critérios foram adotados para garantir a confiabilidade e validade dos julgamentos realizados pelas juízas na composição do banco de vozes.

Todos os voluntários da etapa de validação deveriam seguir os seguintes critérios de elegibilidade: ser fonoaudiólogo, possuir experiência na área de voz, não possuir alteração auditiva autorreferida e/ou diagnosticada e preencher o questionário hospedado *online*, com dados sociodemográficos e julgamento perceptivo-auditivo dos sinais sonoros. A amostra foi composta por 10 fonoaudiólogas juízas com experiência na área de voz na etapa de validação (Tabela 2).

**Tabela 2:** Caracterização sociodemográfica e de formação das juízas fonoaudiólogas

**Variáveis**	**Frequência**	**Porcentagem**
**Grau de Instrução**		
Graduação	5	50%
Mestrado	0	0%
Doutorado	0	0%
Pós-Doutorado	5	50%
**Tempo de Formação**		
Menos de 1 anos	0	0%
De 1 a 5 anos	4	40%
De 6 a 10 anos	0	0%
Mais de 10 anos	6	60%
**Possui Especialização na área de voz**		
Sim	6	60%
Não	4	40%
**Tempo de atuação na área da voz**		
Menos de 1 anos	0	0%
De 1 a 5 anos	4	40%
De 6 a 10 anos	0	0%
Mais de 10 anos	6	60%
**Região do Brasil**		
Sudeste	5	50%
Nordeste	4	40%
Sul	1	10%
**Possui alguma alteração auditiva**		
Sim	0	0%
Não	10	100%

### Materiais

#### Elaboração do EMOVOX-BR

As ferramentas utilizadas foram: o questionário hospedado *online*, com objetivo de levantar dados sociodemográficos dos atores e/ou estudantes de Artes Cênicas, sendo esse composto por 12 itens que abordavam questões como: nome, idade, sexo, estado civil, grau de instrução, data e local de nascimento, endereço, e-mail, telefone, profissão e renda familiar, também foram coletados dados acerca da participação em alguma companhia teatral, o tempo e o período de trabalho, além de investigar se o voluntário possuía smartphone e/ou computador e o sistema operacional utilizado.

A coleta foi realizada em ambiente remoto em momento posterior, agendado após a resposta ao formulário *online*. A plataforma utilizada foi o *Zoom Meeting* de chamada de vídeo, escolhida por sua praticidade e fácil acesso, e por possuir segurança de ponta a ponta dos dados^(18)^. A gravação era feita via computador e smartphone com e sem microfone de todos os voluntários. Ainda, foi utilizado aplicativo Audacity versão 3.0.2, com o uso desta ferramenta todos os sinais foram salvos no formato “wav” para que se mantivesse a melhor qualidade, sem perdas, no computador do pesquisador. Foram coletadas três tarefas de fala: vogal /ε/ sustentada; fala automática com contagem de números de 1 a 10; e fala dirigida compostas por frases de motivação fonéticas que compõem o CAPE-V^(17-19)^. Os áudios selecionados foram os relativos à frase “olha lá o avião azul”, na variação das emoções.

#### Validação do EMOVOX-BR

Esta fase envolveu o preenchimento de um formulário *online*. Esse formulário continha dados sociodemográficos das juízas fonoaudiólogas com experiência na área de voz, sendo esse composto por 12 itens que abordavam o nome, idade, sexo, estado civil, data e local de nascimento, endereço, e-mail, telefone, renda familiar, grau de instrução, também foram coletados dados acerca do tempo de formação, se possuíam especialização na área de voz e alteração auditiva.

Na sequência, as juízas foram instruídas a ouvir 200 áudios nas variadas emoções e registrar as seguintes informações: a emoção identificada (alegria, surpresa, raiva, tristeza, nojo, neutra e medo); a intensidade ou potência com que a emoção foi transmitida (avaliada em uma escala de zero a 10); a valência (positiva, negativa ou neutra); e o parâmetro vocal que consideraram mais relevante para o reconhecimento da emoção (como *pitch*, *loudness*, articulação, velocidade de fala, incoordenação pneumofonoarticulatória, fluência e qualidade vocal). Dos 200 áudios, 182 eram originais e 18 (10%) foram repetições aleatórias, usados para posterior análise de confiabilidade intrajuiz.

### Procedimentos de coleta de dados

#### Elaboração do EMOVOX-BR

Inicialmente a pesquisa foi divulgada por meio de redes sociais. Os voluntários que demonstraram desejo em participar da pesquisa foram informados quanto aos objetivos da pesquisa. Os voluntários receberam instruções sobre as tarefas de fala que precisavam executar e treinar previamente para a simulação das emoções na sessão de gravação, além de ler e concordar com o Termo de Consentimento Livre e Esclarecido (TCLE). O TCLE foi encaminhado também por e-mail com a segunda via assinada pela pesquisadora responsável.

Os voluntários responderam um questionário hospedado no Google Forms. Esse coletou dados sociodemográficos dos atores e estudantes de Artes Cênicas. Após essa coleta inicial, os voluntários recebiam um tutorial com roteiro e procedimentos de gravação e em seguida realizavam o agendamento para a coleta da voz de forma online dos voluntários simulando as emoções. Foram coletadas três tarefas de fala distintas, mencionadas anteriormente, nas variadas emoções.

A seleção dos sinais de áudio para o EMOVOX-BR seguiu critérios metodológicos e de qualidade rigorosos, fundamentados em estudos sobre a coleta de voz online e tarefas de fala^(20,21)^. Baseou-se na utilização de modalidades de fala dirigida, específicas do protocolo CAPE-V, e no método de captura direta via *line in*, ambos reconhecidos por assegurar uma boa relação sinal-ruído (SNR) para registros remotos. Para garantir a clareza e a qualidade dos áudios, todos os sinais foram submetidos a uma análise de SNR, com a seleção restrita aos áudios que apresentaram um SNR igual ou superior a 30dB, conforme padrões da literatura^(22)^.

Estudos prévios demonstraram que a gravação com smartphones é uma opção eficaz e acessível, assegurando que a captura da voz ocorresse com qualidade satisfatória para análises posteriores^(20,21)^. Portanto foram selecionados os áudios coletados por meio da gravação com smartphones, usando a plataforma *Zoom meeting*, motivada pela praticidade e pela alta qualidade oferecida por essa combinação no ambiente remoto.

A plataforma *Zoom* foi selecionada por facilitar o acesso dos participantes e proporcionar uma conexão segura e de fácil utilização. Esse método garantiu a inclusão de voluntários de diversas localidades, bem como a manutenção de SNR ideal, assegurando a fidelidade dos sinais gravados e reduzindo a interferência de ruídos externos. Como resultado, entre os 1.638 áudios coletados, foram escolhidos 182 sinais que atenderam a todos os critérios de qualidade e elegibilidade. Esses áudios representaram de forma fidedigna as emoções simuladas, sendo encaminhados para o julgamento perceptivo-auditivo realizado pelas juízas fonoaudiólogas na etapa de validação.

#### Validação do EMOVOX-BR

Nessa etapa, buscou-se coletar informações sobre o JPA realizado pelas juízas como também os parâmetros de voz e fala que foi importante para o reconhecimento das emoções a partir da voz. As juízas fonoaudiólogas obtiveram acesso ao formulário hospedado no *google forms*. Esse foi subdividido em duas sessões, inicialmente buscou coletar dados sociodemográficos e a segunda sessão foi composta com as vozes dos atores simulando as variadas emoções.

As juízas ouviam atentamente os áudios, avaliavam e registravam suas percepções em relação às solicitações anteriormente descritas, com o objetivo de identificar quais áudios representavam de forma mais precisa cada emoção. Em cada análise, as juízas classificavam a emoção predominante em cada áudio, considerando a intensidade e valência das emoções e os parâmetros vocais mais relevantes para o reconhecimento da emoção transmitida. Esse processo permitiu avaliar a clareza e a consistência emocional de cada registro, que assegurou a representatividade dos áudios na composição do banco de vozes. Cada juíza dedicou, em média, 40 minutos a essa etapa de julgamento perceptivo, considerando o tempo total necessário para avaliar todos os 200 áudios.

### Análise dos dados

Os dados foram tabulados em planilha digital para análise estatística descritiva, por meio de medidas de frequência absoluta e relativa, bem como de tendência central, como médias e desvio padrão, a depender do tipo de variável. Na sequência, foram empregados testes estatísticos inferenciais. As emoções foram consideradas variáveis dependentes. Valência e potência das emoções e parâmetros de voz e fala consideradas independentes, para análise inferencial.

Para identificar as amostras vocais mais representativas para cada emoção, foi realizada análise do grau de confiabilidade intra-áudios e concordância inter-juízes, por meio do teste de concordância de Kappa, que se baseia no número de acertos das emoções propostas em cada amostra vocal, ou seja, em quantas amostras vocais as juízas assinalaram a emoção real simulada pelos atores.

Considerou-se valores de Kappa adequados acima de 0,60, como preconiza a literatura, classificando-se valores entre 0,21 e 0,39 como mínimo; 0,40 e 0,59 fraco; 0,60 e 0,79 moderado; 0,80 – 0,90 forte; acima de 0,90 quase perfeito^(23)^.

O teste Qui-quadrado foi utilizado para verificar a associação entre as características da amostra e o percentual de acerto da emoção, suas valências e parâmetros vocais. Todas as análises foram realizadas por meio do software R versão 4.1.1.1 e utilizou-se o nível de significância de 5%.

## RESULTADOS

### Elaboração do EMOVOX-BR

Foram coletados 1.638 sinais sonoros produzidos por atores profissionais e em formação nativos do PT-BR. Desses, foram selecionados 182 áudios para serem avaliados e 39 sinais sonoros para compor o EMOVOX-BR. Desses, 24 áudios são de vozes masculinas e 15 áudios de vozes femininas. A maior parte dos sinais selecionados representam a emoção surpresa e a menor parte a emoção nojo (Figura 1).

**Figura 1:** Processo de Seleção áudios para o julgamento perceptivo-auditivo do EMOVOX-BR

Todos os 39 áudios que compõem o EMOVOX-BR apresentaram confiabilidade superior a 0,7, valor considerado satisfatório de acordo com o preconizado. O total de 24 áudios (61%) obtiveram concordância quase perfeita segundo análise das juízas (Tabela 3), ou seja, as amostras representam de fato a emoção simulada.

**Tabela 3:** Descrição dos áudios e valor da confiabilidade no KAPPA

**Variáveis**	**Sexo**	**Emoção**	**Valor de concordância KAPPA**
Áudio 05	Masculino	Surpresa	0,855
Áudio 07	Masculino	Neutra	0,799
Áudio 11	Masculino	Raiva	0,711
Áudio 12	Masculino	Surpresa	0,808
Áudio 14	Masculino	Neutra	0,799
Áudio 15	Masculino	Alegria	1,0
Áudio 18	Masculino	Raiva	1,0
Áudio 21	Masculino	Neutra	1,0
Áudio 26	Feminino	Surpresa	0,714
Áudio 28	Feminino	Neutra	0,835
Áudio 30	Masculino	Medo	0,855
Áudio 33	Masculino	Surpresa	0,753
Áudio 38	Feminino	Tristeza	1,0
Áudio 45	Masculino	Tristeza	0,879
Áudio 52	Masculino	Tristeza	1,0
Áudio 54	Masculino	Surpresa	0,902
Áudio 56	Masculino	Neutra	0,822
Áudio 80	Masculino	Tristeza	0,855
Áudio 81	Masculino	Raiva	0,899
Áudio 82	Masculino	Surpresa	0,783
Áudio 105	Masculino	Neutra	0,877
Áudio 121	Feminino	Medo	1,0
Áudio 123	Feminino	Raiva	0,799
Áudio 127	Masculino	Alegria	0,835
Áudio 128	Masculino	Medo	0,893
Áudio 132	Masculino	Nojo	0,711
Áudio 137	Masculino	Raiva	0,714
Áudio 138	Masculino	Surpresa	0,783
Áudio 139	Masculino	Nojo	1,0
Áudio 142	Feminino	Medo	0,713
Áudio 149	Feminino	Medo	0,855
Áudio 150	Feminino	Tristeza	1,0
Áudio 151	Feminino	Raiva	0,799
Áudio 154	Feminino	Neutra	0,825
Áudio 157	Feminino	Tristeza	1,0
Áudio 165	Feminino	Raiva	1,0
Áudio 178	Feminino	Tristeza	0,714
Áudio 180	Feminino	Surpresa	0,808
Áudio 141	Feminino	Alegria	0,771

### Validação do EMOVOX-BR

O percentual de acerto das emoções na avaliação das juízas foi superior a 70%, configurando um alto índice de reconhecimento das emoções a partir da voz nos áudios que compõem o banco EMOVOX-BR, isto é, as juízas identificaram corretamente a emoção simulada pelos atores (Tabela 4).

**Tabela 4:** Percentual de acerto das variadas emoções por juízas Fonoaudiólogas

**Emoção**	**Frequências**	**Percentual de acerto**	**P-Valor**
Neutra	7	84,4%	0,44^*^
Raiva	7	82,6%	
Nojo	2	75%	
Surpresa	7	73,8%	
Alegria	3	73,3%	
Tristeza	7	72,5%	
Medo	5	71,3%	

Teste Qui-quadrado de Pearson

* Significância p<0,05

A Tabela 5 apresenta os achados quanto a percepção da valência atribuída pelas juízas para as emoções avaliadas nos áudios. As emoções que foram definidas como de valência positiva: alegria e surpresa; de valência negativa: medo, tristeza, raiva e nojo; e de valência neutra: neutra. A taxa de acerto aumenta quando a avaliação é pela valência, com valores acima de 80%. Na Tabela 5 observou-se a identificação dos parâmetros de voz e fala assinalados como mais importantes no reconhecimento das emoções apresentada pelas juízas no JPA.

**Tabela 5:** Descrição da identificação dos parâmetros **de valência e de voz e fala** na variação das emoções na emissão de vozes de acordo com as impressões de juízas fonoaudiólogas

**Variáveis**	**Emoção**														**P-valor**
	**Alegria**		**Medo**		**Tristeza**		**Raiva**		**Surpresa**		**Nojo**		**Neutra**		
	**n**	**%**	**n**	**%**	**n**	**%**	**n**	**%**	**n**	**%**	**n**	**%**	**n**	**%**	
**Valência**															
Positiva	27	90%	5	8,3%	1	1,3%	2	2,9%	70	85,7%	4	20%	2	2,9%	0,0001*
Negativa	3	10%	51	85%	61	76,3%	63	90%	9	11,3%	16	80%	12	17,1%	0,0001*
Neutra	0	0%	4	6,7%	18	22,5%	5	7,1%	1	1,3%	0	0%	56	80%	0,0001*
**Parâmetros de voz e Fala**															
*Pitch*	21	70%	30	51,7%	49	61,3%	40	57,1%	62	77,5%	13	65%	36	51,4%	0,003*
*Loudness*	11	36,7%	28	46,7%	28	35%	35	50%	31	38,8%	5	25%	17	24,3%	0,003*
Articulação	4	13,3%	4	6,7%	3	3,8%	17	24,3%	10	12,5%	4	20%	5	7,1%	0,0002*
Velocidade F	11	36,7%	18	30%	33	41,3%	33	47,1%	28	36,3%	70	35%	24	34,3%	0,004^*^

Teste Qui-quadrado de Pearson

* Significância p<0,05

Observa-se que o *pitch* foi o parâmetro mais citado para identificar todas as emoções básicas: alegria, medo, tristeza, raiva, surpresa e nojo. A *loudness* foi importante para reconhecer a emoção raiva. Já articulação, velocidade de fala, ICPFA, qualidade vocal e fluência não foram parâmetros assinalados como importantes para a determinar a classificação das emoções apresentadas, em todas as emoções com resultado inferior a 50%.

## DISCUSSÃO

A elaboração e o processo de validação do pioneiro EMOVOX-BR surgiu da escassez de banco de vozes com variações emocionais no PT-BR, além do ineditismo de passar pelo processo de validação por juízes fonoaudiólogos com experiência na área de voz. O EMOVOX-BR foi composto por 39 áudios, dos quais 24 são de vozes masculinas e 15 de vozes femininas, abrangendo as emoções alegria, surpresa, raiva, tristeza, nojo, neutra e medo. A seleção das seis emoções básicas, reconhecidas por “Big-Six”, acrescida da emissão neutra, que diz respeito ao sinal de áudio que não tem predomínio de nenhuma das emoções, foi direcionada a partir de estudos prévios^(18,20,21,24).^

Atualmente, diversos bancos de vozes incorporam variações emocionais e abrangem populações de atores em diferentes idiomas e culturas ao redor do mundo, porém poucos estão disponíveis para acesso aberto à comunidade científica^(11-14)^. Vale destacar que a maioria desses bancos foi desenvolvida com amostras de falantes adultos, sendo que apenas dois incluem vozes infantis, dos quais um é específico para variações de estresse.

Alguns desses bancos compartilham uma estrutura de desenvolvimento semelhante, que inclui características como seleção de participantes, acessibilidade e tipos de emoções analisadas^(12-14)^. No entanto, essas bases internacionalmente reconhecidas foram criadas principalmente para investigar as variações acústicas da fala, com pouca ênfase no JPA por especialistas^(11)^. Além disso, são bases internacionais e não incluem amostras de falantes do PT-BR.

Alguns parâmetros identificados na voz e fala podem ser entendidos como um conjunto de fenômenos suprassegmentais, como a velocidade de fala, ritmo no aspecto temporal, organização melódica (acento, melodia, entonação) e intensidade (volume, força) presentes na fala^(25,26)^, bem como os parâmetros psicoacústicos, *pitch* e *loudness*.

O JPA é um recurso tradicional utilizado na prática clínica da área de voz, porém, depende da experiência do avaliador^(27-29)^. O curso de Fonoaudiologia prepara para o julgamento dos parâmetros vocais, principalmente os relativos à qualidade vocal. A literatura indica, contudo, que além do treinamento intenso, outros fatores são fundamentais para aumentar a confiabilidade dos juízes na percepção auditiva, como a exposição a uma ampla variedade de vozes, a qualidade e diversidade do material vocal utilizado, o tipo de pergunta formulada e a experiência prévia com diferentes nuances emocionais^(28,29)^.

O percentual de acerto das emoções avaliadas pelas juízas nos sinais que compõem o EMOVOX-BR indica que as amostras vocais são representativas e eficazes na expressão das emoções. A emoção surpresa, seguida de tristeza e raiva, apresenta o maior número de áudios selecionados para o EMOVOX-BR, enquanto o nojo possui o menor número de áudios. As emoções com o maior percentual de acerto foram raiva e neutra, ao passo que o medo obteve o menor índice de acerto. Este achado é confirmado pela literatura, que destaca a raiva como a emoção de maior impacto na identificação pelo interlocutor, exige mais energia para sua produção e pode associar-se a alterações no posicionamento da laringe, velocidade de fala e intensidade vocal^(10,20,27)^.

Houve uma taxa de acerto alta no reconhecimento das emoções a partir da voz pelo grupo de profissionais com experiência na área de voz, onde obtiveram valores superiores a 70% em todas as emoções (alegria, medo, tristeza, raiva, surpresa, nojo e neutra). Estudo anterior^(30)^ sobre detecção de baixa e alta ansiedade na análise de vozes com leigos e profissionais generalistas obtiveram taxa de acerto em torno de 50%, portanto, a experiência das juízas pode ser um achado que influencie a taxa de acerto.

A valência das emoções, analisada neste estudo, refere-se ao caráter positivo, negativo ou neutro da emoção expressa^(31)^. Além das valências positiva e negativa, foi considerada também a valência neutra, caracterizada pela ausência de uma emoção específica na emissão vocal. As juízas classificaram as emoções como de valência positiva (alegria e surpresa), negativa (medo, raiva, tristeza e nojo) e neutra (neutra). Observa-se que as mudanças de emoções e valências influenciam a expressividade vocal, sendo que a repetição de padrões emocionais negativos pode resultar em efeitos fisiológicos, como ressecamento da mucosa oral, que pode interferir na fala, com repercussão no controle muscular oral e na articulação dos fonemas, além de possivelmente provocar instabilidade da qualidade vocal^(7,32)^. Dessa forma, o estado emocional afeta a atividade comunicativa como um todo, e não apenas a voz^(30)^.

A identificação dos parâmetros de voz e fala foi essencial para a caracterização das emoções nos áudios, segundo o JPA realizado pelas juízas fonoaudiólogas. Observou-se que o *pitch* foi o parâmetro mais frequentemente associado às emoções alegria, medo, tristeza, raiva, surpresa, nojo e neutra, enquanto o loudness destacou-se na emoção raiva. Outros parâmetros, como articulação, velocidade de fala, fluência, qualidade vocal e incoordenação pneumofonoarticulatória (ICPFA), não foram considerados relevantes para a classificação das emoções. Esse achado pode ser explicado pela ausência de alterações vocais nos falantes, cujos sinais não apresentavam características de disfonia que poderiam comprometer a inteligibilidade e a expressividade vocal.

É importante destacar que o *pitch* e a *loudness* são parâmetros psicoacústicos, pois dependem da percepção do ouvinte, com *pitch* associado à frequência do som e *loudness* à intensidade percebida. No contexto deste estudo, esses parâmetros ajudam a identificar nuances emocionais, que pode permitir explorar a carga emocional da voz além da qualidade vocal, o que é essencial para capturar a complexidade da expressividade^(33)^.

De modo geral, os parâmetros de *pitch* e *loudness* reafirmaram-se como fundamentais para a identificação das emoções a partir da voz^(7-9,32)^. Estudos indicam que, em diferentes línguas, a emoção alegria é frequentemente caracterizada por um *pitch* elevado, *loudness* forte e pausas breves. A emoção raiva, por sua vez, apresenta *pitch* elevado, *loudness* mais fraca em homens e forte em mulheres, além de uma velocidade de fala mais rápida em mulheres. Já a emoção nojo é comumente expressa por um *pitch* grave, *loudness* fraca e velocidade de fala reduzida, que reflete sua intensidade e valência negativa^(34,35)^.

Portanto, juízes experientes puderam reconhecer as emoções a partir da voz em falantes nativos do PT-BR e perceber características comuns nas variações emocionais, por meio do JPA. Esses achados contribuem para a comunidade científica uma vez que possuem áudios testados e validados com alta confiabilidade, que podem auxiliar na criação sistemas de reconhecimento de padrões, e na clínica fonoaudiológica podem auxiliar no aprofundamento de estudos futuros sobre estratégias de autorregulação, mudança de comportamento, controle vocal e gerenciamento das emoções.

Mais possibilidades se abrem numa perspectiva voltada ao aprimoramento vocal, psicodinâmica e intervenções voltadas aos profissionais da voz, como composição de personagens junto a atores. Além disso, confirma que a voz é um sinal biológico de interesse interdisciplinar, coletado de forma não invasiva, que abre diversas possibilidades para estudos do reconhecimento de padrões com utilização de modelos estatísticos de ponta para discriminar ou predizer diversos contextos tecnológicos, sociais, culturais e de saúde.

## CONCLUSÃO

O Banco de Vozes Brasileiro nas Variadas Emoções (EMOVOX-BR) foi desenvolvido e validado para representar as emoções alegria, medo, tristeza, raiva, surpresa, nojo e neutra em falantes do PT-BR. Composto por 39 áudios de alta confiabilidade quanto a qualidade, identificação e intensidade das emoções, o banco obteve alta taxa de acerto na avaliação das juízas com experiência na área de voz. O parâmetro *pitch* destacou-se na identificação de todas as emoções, enquanto a *loudness* foi particularmente relevante para identificar a emoção raiva. Dessa maneira, o EMOVOX-BR foi validado, fato que evidencia sua eficácia na representação de diferentes emoções e suas características distintivas, perceptíveis por avaliadores especializados.
